# CBCT analysis of the tissue thickness at immediate implant placement with contour augmentation in the maxillary anterior zone: a 1-year prospective clinical study

**DOI:** 10.1186/s40729-021-00344-9

**Published:** 2021-07-06

**Authors:** Yuya Fujita, Tamaki Nakano, Shinji Ono, Takuya Shimomoto, Keiichiro Mizuno, Hirofumi Yatani, Shoichi Ishigaki

**Affiliations:** Department of Fixed Prosthodontics, Osaka University Graduate School of Dentistry, 1-8 Yamadaoka, Suita, Osaka, 565-0871 Japan

**Keywords:** Immediate implant placement, Guided bone regeneration, Connective tissue graft, Bone and soft tissue change, Cone-beam CT, Prospective study, Anterior maxilla, Preoperative and postoperative

## Abstract

**Background:**

Immediate implant placement with simultaneous contour augmentation such as guided bone regeneration (GBR) or connective tissue grafting (CTG) has been widely performed. However, few prospective studies have evaluated both peri-implant bone and soft tissue changes between the preoperative and postoperative periods. The purpose of this study is to quantify the horizontal dimensional changes of the facial bone and soft tissue following immediate implant placement with contour augmentation.

**Material and methods:**

Twenty patients who underwent immediate implant placement in the anterior maxilla received GBR and CTG (test group) or GBR only (control group). Cone-beam computed tomography (CBCT) scans were taken preoperatively and 1 year after the definitive prosthesis connection, and then, they were superimposed. On the CBCT images of the two stages, the horizontal distance from the implant platform to the facial bone surface (BW) and the horizontal soft tissue width (GW) were measured at the implant platform level and 2 mm apical to the implant platform level. The sum of BW and GW (=TW) was used to assess the facial mucosal contour.

**Results:**

BW decreased significantly from preoperative to 1 year after prosthesis connection with a mean decrease of 0.47 mm (*P* =0.021) in the control group and a mean decrease of 0.50 mm (*P* = 0.019) in the test group at the implant platform level. GW increased significantly with a mean increase of 1.37 mm (*P* =0.005) in the test group at the implant platform level. TW decreased significantly with a mean decrease of 0.46 mm in the control group (*P* =0.049) but increased significantly with a mean increase of 0.87 mm in the test group (*P* =0.005) at the implant platform level.

**Conclusions:**

Immediate implant placement with CTG showed a soft tissue gain of 1.37 mm compensated for bone resorption, thus still preserving the preoperative mucosal contour. CTG should be performed with immediate implant placement in cases where preoperative mucosal contours need to be maintained.

## Background

Both functional recovery and esthetic restoration are essential in implant treatment in the maxillary anterior zone. Harmony between the peri-implant tissue and the remaining natural teeth must be achieved and maintained over the long term. Sufficient thickness of the bone and soft tissue around the implant is reportedly necessary to prevent recession of the soft tissues after treatment and ensure long-term maintenance of esthetics [1].

The concept of placing an implant into the tooth socket immediately following extraction has been widely accepted because it prevents post-extraction bone resorption and preserves the initial mucosal contour [2, 3]. However, clinical studies have indicated that facial bone wall resorption is likely to occur even when an implant is placed immediately after tooth extraction. Also, there is a high risk of gingival recession after treatment, especially in the maxillary anterior teeth, which typically have a thin facial bone or labial bony defects [4–6]. Therefore, simultaneous contour augmentation such as guided bone regeneration (GBR) or connective tissue grafting (CTG) is required in most immediate placement cases to establish a facial bone and soft tissue of sufficient thickness and to minimize gingival recession and volume loss of peri-implant tissue. In particular, recent studies have reported the effectiveness of placing a soft tissue graft in the esthetic zone to acquire more stable peri-implant mucosal soft tissues [7–9].

In recent years, several studies have been conducted to evaluate the dimensional changes in the peri-implant bone or soft tissues after implant placement, using probes, dental radiographs, or cone-beam computed tomography (CBCT) [10–12]. However, few prospective studies have evaluated both peri-implant bone and soft tissue changes simultaneously following immediate implant placement. The lack of such studies is because no method has been established to accurately evaluate the dimensional changes of peri-implant tissues between the preoperative and postoperative periods. Although bone and soft tissues can reportedly be evaluated prior to and after surgery by direct measurement with a periodontal probe or endodontic needle, the reliability and validity of such measurements are inferior, and it is impossible to evaluate the bone and soft tissue at the same time [13–15]. Although the preoperative mucosal contours can be evaluated by superimposing of dental casts, it is impossible to evaluate the bone and soft tissue separately [16, 17]. Therefore, the augmentation induced by changes in the bone and soft tissue between the preoperative and postoperative periods remains unclear, and no specific criteria for choosing the most appropriate surgical procedure have been established. In this study, we devised a method to create a cross-sectional image based on the jaw bone by superimposing CBCT data, unlike the conventional method based on the implant body [11, 18]. Additionally, bone and soft tissue can be evaluated on CBCT images by displacing the lips before performing the CBCT scan [19, 20]. As a result, we compared the same cross-sectional image preoperatively and postoperatively and accurately evaluated the dimensional changes in bone and soft tissues following implant placement with augmentation.

The purpose of this study was to quantify the horizontal dimensional changes in the facial bone and soft tissue following immediate implant placement with contour augmentation in the maxillary anterior zone.

## Material and methods

### Patient selection

From April 2013 to September 2017, 20 patients who visited the Department of Fixed Prosthodontics, Osaka University Dental Hospital, Osaka, Japan, participated in this prospective clinical study. This study included all eligible consecutive patients who underwent immediate implant placement in central or lateral maxillary incisors during the observation period according to the following inclusion and exclusion criteria.

#### Inclusion criteria

1. Good oral hygiene, defined as a full-mouth plaque score ≦25%
2. Sufficient native bone around the failing tooth to achieve primary stability of the implant
3. Fixed prosthesis connected to the implant

#### Exclusion criteria

1. A history of smoking or diabetes
2. Acute inflammation or infection such as pus and fistula around the treatment site at the time of implant placement
3. A labial bony defect around the failing tooth mesiodistally greater than the implant diameter or vertically >4mm apical to the implant platform level

This study was approved by the ethical committee of Osaka University Dental Hospital and Osaka University Graduate School of Dentistry.

### Surgical procedures

After administering 2% lidocaine solution for local anesthesia, a mucoperiosteal flap was raised, and tooth extraction was performed to avoid damaging the facial alveolar bone. Extraction sockets were debrided with a bone curette. A NobelActive® implant (Nobel Biocare, Gothenburg, Sweden) was placed at ideal positioning of the prospective implant prosthesis and a depth of 4 mm apical to the most apical aspect of the prospective clinical crown with the help of a surgical template, and a cover screw was connected. Simultaneous GBR procedures were performed on the implant’s facial aspects using deproteinized bovine bone (Bio-Oss®; Geistlich Pharma, Wolhusen, Switzerland), and this bone substitute material was covered with a resorbable collagen membrane (Bio-Gide®; Geistlich Pharma, Wolhusen, Switzerland). Following the apical release of the periosteum tension, the flap was coronally advanced and sutured using 5-0 monofilament sutures (Ethilon 698G, Shofu, Kyoto, Japan). Each patient received antibiotics (300 mg Cefcapene pivoxil hydrochloride hydrate, Flomox, Shionogi, Osaka, Japan) and was prescribed 400 mg of loxoprofen (Loxonin; Daiichi Sankyo Healthcare, Tokyo, Japan) for pain control. The sutures were removed 14 days after surgery.

**Fig. 1:**
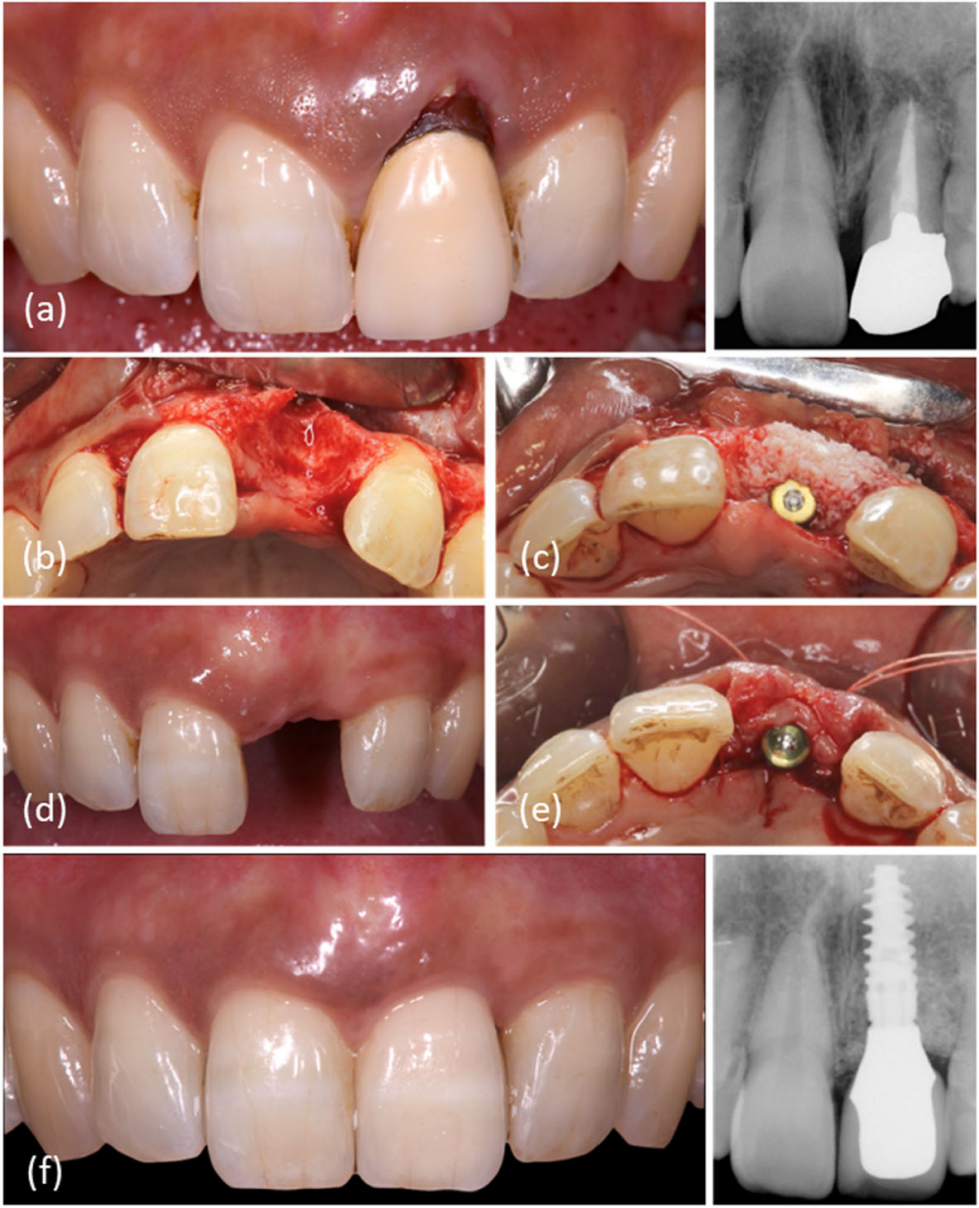
The surgical and prosthesis procedures of the test group. **a** Buccal view of the preoperative situation. **b** A mucoperiosteal flap was raised, and the tooth was extracted. **c** Deproteinized bovine bone was inserted into the implant-socket gap. **d** Buccal view before the second surgery. **e** Connective tissue was placed in a supraperiosteal envelope flap. **f** Buccal view after definitive prosthesis connection

After 4 months, all patients proceeded with the second surgery to connect a 5-mm height healing abutment. In patients who received CTG (test group), connective tissue was collected from the palatal mucosa and placed in a supraperiosteal envelope flap (Fig. 1).

All the surgical procedures were always performed by two experienced surgeons qualified as specialists with the Japanese Society of Oral Implantology.

### Prosthesis procedures

After a healing period of 2 weeks, impressions were taken, and a screw-retained provisional crown was connected. In a situation in which the peri-implant gingival level is coronal to the contralateral tooth, the emergence profile or facial contour of the provisional restoration is modified to recede the gingival margin to recede to achieve symmetry with the contralateral tooth. After 2 months of function, impressions were taken for the definitive prosthesis using individualized impression coping. A screw-retained zirconia-based prosthesis (NobelProcera; Nobel Biocare) was connected and tightened to the implants using a torque wrench set to 35 Ncm (Fig. 1).

### CBCT image acquisition

CBCT images were acquired with an Alphard 3030 instrument (Asahi Roentgen, Kyoto, Japan). CBCT was taken preoperatively (T0) and 1 year after the definitive prosthesis (T1). The upper lip was displaced in the oral vestibule by placing a dry cotton roll before taking the scans to visualize the peri-implant soft tissue in the CBCT images.

**Fig. 2:**
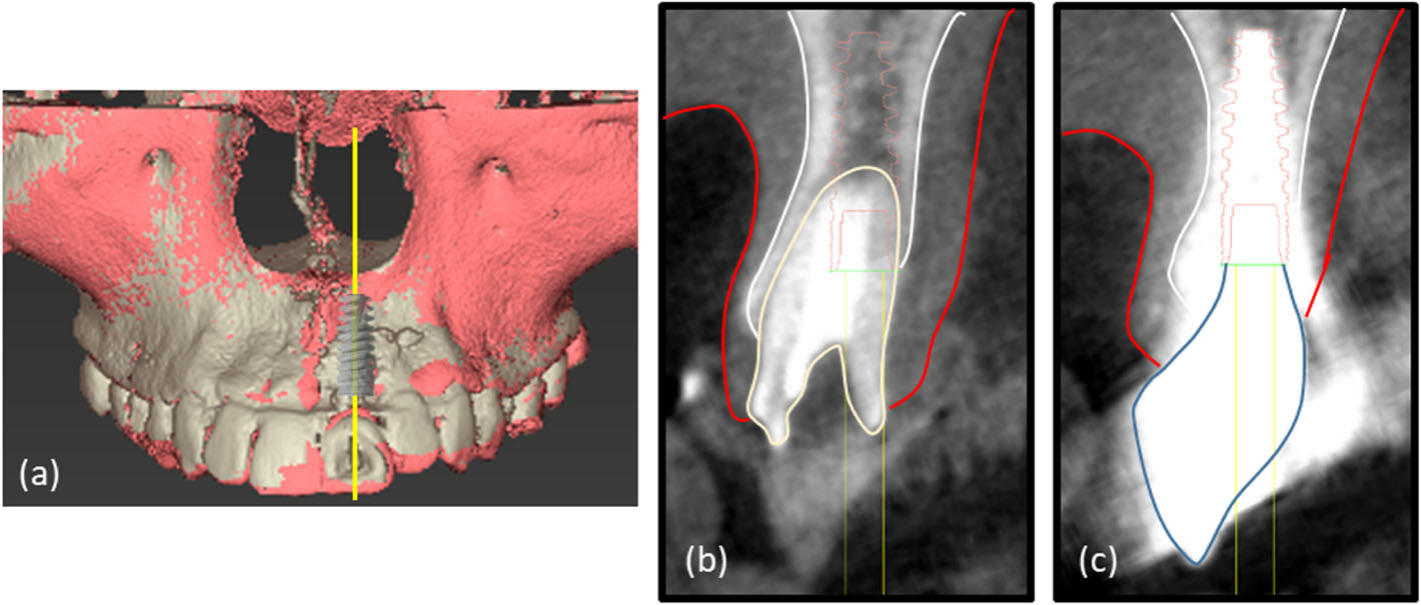
CBCT image acquisition preoperatively (T0) and 1 year after connection of the definitive prosthesis (T1). **a** Superimposition of 3D maxillary models obtained from cone-beam computed tomography scans at T0 and T1. **b** Buccopalatal cross-sectional images at T0 acquired with reference to the long axis of a virtual implant placed in the exact position where the actual implant would be. **c** Buccopalatal cross-sectional images at T1 acquired with reference to the long axis of the implant

Three dimensional (3D) maxillary models were then obtained from both T0 and T1 scans by reconstructing DICOM data and superimposing them using a software (coDiagnostiX, Dental Wings, Montreal, Canada). Reference points for the superimposition of these 3D models were set to the left and right sides of the lower orbital pits and the sternal process of the maxilla. Additionally, the implant’s long axis on the superimposed 3D models was used as a reference to acquire the T0 and T1 buccopalatal cross-sectional images. A virtual implant could then be placed on the T0 scans in the exact position of the actual implant after surgery (Fig. 2).

CBCT scans at the definitive prosthesis connection and 1 year later were extracted from 10 previous patients who have passed 1 year or more after treatment completion to assess the accuracy of the superimposition of the 3D maxillary models. The 3D models obtained from the CBCT scans were superimposed, and the 3D deviation was calculated at the implant base and apex using the coDiagnostiX software. At the implant base, the mean deviation was 0.13±0.05 mm, and at the implant apex, the mean deviation was 0.07±0.04 mm. The values shown above indicate average errors when superimposing 3D maxillary models of T0 and T1 in the present study. The superimposition of the 3D maxillary models may be more accurate than the model and CBCT because the jawbone area is not affected by metallic artifacts of restorations in the dental arch [21].

### Horizontal hard and soft tissue measurements

**Fig. 3:**
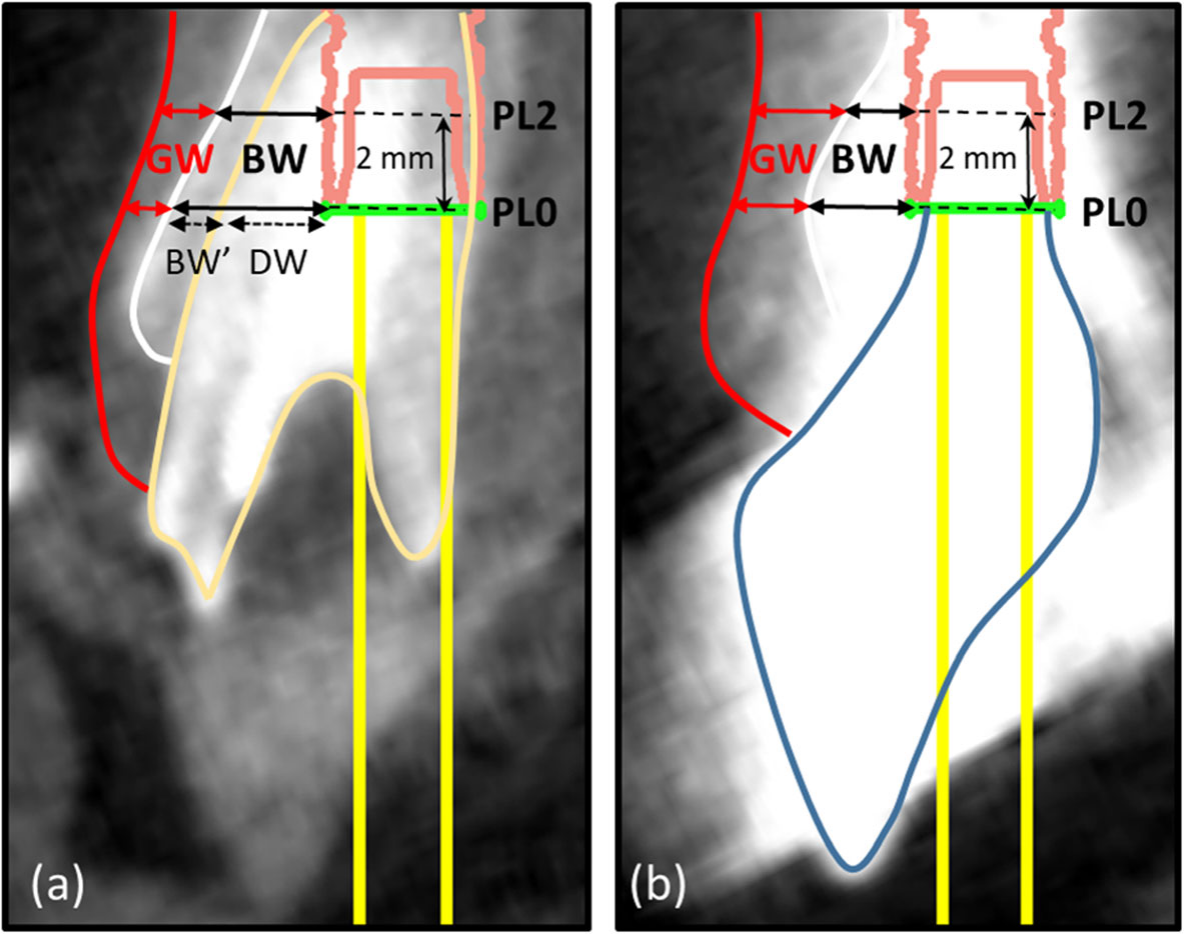
Measurement on buccopalatal cross-sectional images preoperatively and 1 year after connection of the definitive prosthesis. **a** Virtual implant models were used as the measurement reference preoperatively. The horizontal distance from the implant surface to the facial bone surface (BW) and the horizontal width of the soft tissue (GW) were measured at the implant platform level (PL0) and 2 mm apical to the implant platform (PL2). The implant-socket gap (the distance from the virtual implant surface to the root surface) (DW) and the horizontal width of the facial alveolar bone (BW’) were measured at PL0. **b** The horizontal width of bone (BW) and soft tissue (GW) were measured at PL0 and PL2 1 year after connection of the definitive prosthesis

After the superimposition of the 3D maxillary models, the horizontal distance from the implant surface to the facial bone surface (or the root surface if there was a labial bony defect at the measurement level preoperatively, BW) and the horizontal width of the soft tissue (GW) were measured at the implant platform level (PL0) and 2 mm apical to the implant platform level (PL2) on both T0 and T1 scans (Fig. 3). Because there was no implant at T0, virtual implant models acquired by superimposition were used as the measurement reference on the T0 scan. The sum of BW and GW (=TW) was used to assess the mucosal contour.

Additionally, the implant-socket gap (the distance from the virtual implant surface to the root surface) (DW) and the horizontal width of the facial alveolar bone (BW’) were measured at PL0 on the T0 scans (Fig. 3). DW was measured to assess where the implant was placed buccopalatal to the extraction socket; BW’ was measured to assess the preoperative initial alveolar bone thickness facially to the implant.

The reliability and reproducibility of these measures on CBCT scans have already been shown [11, 18].

### Data analysis

Statistical analysis of all collected data was performed with SPSS ver. 23 (IBM, Tokyo, Japan).

Measurements on the T0 and T1 scans were compared using the Wilcoxon signed-rank test. Statistical significance was accepted at α< .05.

## Results

In total, 20 immediately placed implants in 20 patients were included per group, consisting of 10 patients in the control group and 10 in the test group. The baseline patient characteristic and initial tissue thickness in the two groups are described in Table 1. There were no statistically significant differences in age, sex, treatment site, or initial bone and soft tissue thickness between the groups. No patients dropped out at the 1-year follow-up, and all implants were integrated, resulting in a 100% implant survival.

**Table 1:** Patient demographics and initial horizontal bone and soft tissue thickness per study group

		Control group	Test group	*P-*value
Sex^a^ (Male:female)		3:7	2:8	0.606
Age (years)^b^		55.2 ± 18.1	48.2 ± 13.7	0.315
Site^a^ (central incisor:lateral incisor)		5:5	5:5	1.000
BW at T0 (mm)^b^	PL0	2.28 ± 0.76	2.71 ± 0.42	0.123
	PL2	2.03 ± 0.75	2.36 ± 0.42	0.436
GW at T0 (mm)^b^	PL0	1.43 ± 0.46	1.19 ± 0.21	0.063
	PL2	1.53 ± 0.26	1.37 ± 0.26	0.280

Values shown are the number of patients or a mean with standard deviation

^a^χ^2^ test, significance level of 0.05 was used

^b^Mann-Whitney *U* test, significance level of 0.05 was used

### Facial hard tissue changes

BW decreased significantly from T0 to T1 with a mean decrease of 0.47 mm at PL0 (*P* =0.021) in the control group and a mean decrease of 0.50 mm at PL0 (*P* = 0.019) in the test group. Nevertheless, a mean of 1.81 mm at PL0 and 1.97 mm at PL2 in the control group and 2.21 mm at PL0 and 2.26 mm at PL2 in the test group horizontal bone thickness was present facially to the implant on the T1 scan, respectively (Table 2).

**Table 2:** Horizontal facial hard and soft tissues and mucosal contour changes following immediate implant placement

Measurement		Preoperative		1 year after prosthesis connection		*P*-value^*^
		Median (Min; Max) mm	Mean (SD) mm	Median (Min; Max) mm	Mean (SD) mm	
Test group (*n*=10)						
BW	PL0	2.85 (0.6; 3.0)	2.71 (0.42)	1.90 (1.3; 3.3)	2.21 (0.57)	0.019
	PL2	2.40 (1.6; 3.1)	2.36 (0.42)	2.10 (1.5; 3.4)	2.26 (0.57)	0.474
GW	PL0	1.25 (0.8; 1.5)	1.19 (0.21)	2.60 (2.0; 3.2)	2.56 (0.30)	0.005
	PL2	1.35 (0.9; 1.7)	1.37 (0.26)	2.65 (2.1; 2.8)	2.57 (0.24)	0.005
TW	PL0	3.85 (3.1; 4.8)	3.90 (0.46)	4.80 (3.6; 5.7)	4.77 (0.61)	0.005
	PL2	4.00 (2.6; 4.3)	3.73 (0.57)	4.80 (3.6; 6.0)	4.83 (0.68)	0.005
Control group (*n*=10)						
BW	PL0	2.30 (1.2; 3.9)	2.28 (0.76)	1.75 (1.2; 2.7)	1.81 (0.40)	0.021
	PL2	2.00 (0.9; 3.9)	2.03 (0.75)	2.00 (1.0; 3.0)	1.97 (0.53)	0.720
GW	PL0	1.25 (0.9; 2.3)	1.43 (0.45)	1.35 (0.9; 2.2)	1.44 (0.40)	0.887
	PL2	1.50 (1.1; 1.9)	1.53 (0.26)	1.60 (1.0; 2.1)	1.56 (0.34)	0.524
TW	PL0	4.05 (2.1; 5.1)	3.71 (0.96)	3.25 (2.1; 4.3)	3.25 (0.68)	0.049
	PL2	3.65 (2.3; 5.4)	3.56 (0.85)	3.50 (2.1; 4.7)	3.53 (0.82)	0.905

^*^Wilcoxon signed-rank test; a significance level of 0.05 was used.

*PL0* Implant platform level, *PL2* 2 mm Apical to the implant platform level, *BW* The horizontal distance from the implant surface to the facial bone surface, *GW* The horizontal width of the soft tissue, *TW* The sum of BW and GW

### Facial soft tissue changes

There were no significant differences in GW from T0 to T1 in the control group. Whereas, GW increased significantly from T0 to T1 in the test group, with a mean increase of 1.37 mm at PL0 and 1.20 mm at PL2 (*P* =0.005). As a result, a mean of 2.56 mm at PL0 and 2.57 mm at PL2 horizontal soft tissue thickness was present facially to the implant on the T1 scan, respectively (Table 2).

### Mucosal contour changes

TW decreased significantly by 0.46 mm at PL0 (*P* = 0.049) in the control group, whereas a significant TW increase of 0.87 mm at PL0 (*P* = 0.005) and 1.10mm at PL2 (*P* = 0.005) was observed in the test group (Table 2).

### Horizontal implant–socket gap size and initial facial bone thickness at the preoperative stage

In the case with a labial bony defect at PL0, BW’ was set to 0. On the T0 scan, BW’ was 0.61 mm, and DW was 1.89 mm at PL0 (Table 3).

**Table 3:** Horizontal initial bone thickness (BW’) and the gap distance between the extraction socket and implant (DW) at the preoperative stage

Measurement	Preoperative	
	Median (Min; Max) mm	Mean (SD) mm
DW	1.95 (0.6; 3.0)	1.89 (0.74)
BW’	0.60 (0; 2.0)	0.61 (0.60)

*DW* The distance from the implant surface to the root surface, *BW’* The horizontal width of the facial alveolar bone

## Discussion

In this study, a mean bone thickness of 1.81 mm at PL0 and 1.97 mm at PL2 in the control group and 2.21 mm at PL0 and 2.26 mm at PL2 in the test group was present facial to the implants at the definitive prosthesis connection period. These results suggest that sufficient bone thickness could be acquired with immediate implant placement and delayed provisionalization in both groups [1]. However, in the control group, TW decreased significantly from T0 to T1 at PL0 because of the decrease in BW. The soft tissue thickness (GW) in the control group remained stable following immediate placement, leading to the assumption that mucosal contour loss is mostly related to underlying facial hard tissue resorption. However, in the test group, the decrease in BW was compensated for by the increase in GW gained by CTG, resulting in a significant increase in TW at both PL0 and PL2.

The soft tissue thickness increased significantly with CTG; the mean soft tissue gain was 1.37 mm at PL0 and 1.20 mm at PL2. Previous studies have shown that the thickness of the palatal mucosa of Asians is 2.0 to 3.7 mm, and CTG results in a soft tissue thickness increase of 0.92–1.40 mm as measured with an endodontic needle or ultrasonic device [22–24]. The results obtained from this study are also in line with the previous reports.

Numerous reports have described the superiority and inferiority of immediate implant placement. Multiple studies have shown that immediate implant placement can lead to facial mucosal recessions [25, 26]. On the other hand, placing an implant simultaneously as tooth extraction allows a decrease in the number of surgical procedures and treatment time and minimizes bone and soft tissue resorption after tooth extraction and maintains the preoperative mucosal contour [2, 3]. Postoperative bone resorption is suppressed by combining bone augmentation with immediate implant placement [27]. In the present study, GBR was conducted in all patients simultaneously as immediate implant placement using deproteinized bovine bone mineral (DBBM) granules and resorbable membranes to achieve contour augmentation and prevent bone resorption. Although DBBM granules have a low substitution rate and were used to fill the implant-socket gap to help maintain the dimensions of the facial wall, a mean of 0.49 mm of horizontal hard tissue resorption at PL0 was observed. This result is probably due to the resorption of the original facial bundle bone along with tooth extraction. Particularly in the maxillary anterior region, where the initial facial bone thickness is thin, and most of it is composed of bundle bones attached to the natural tooth through the periodontal ligament. It has been reported that facial bone thickness is less than 1.0mm in most cases and even 0.5mm or less in half of the sites [28]. Therefore, bone resorption is likely to occur after tooth extraction within a few weeks [6, 29]. In the present study, the initial bone width facially to the implant was 0.61mm, suggesting that most original facial bones must have been resorbed during 1 year of follow-up. This result could mean that the newly augmented bone functioned as a new facial plate.

A previous study showed that immediate implant placement is contraindicated if there is a dehiscence bone defect facially to the implant [4]. Otherwise, preoperative labial bony defects can be reconstructed by simultaneous bone augmentation even if a dehiscence bone defect is present at implant placement [27, 30, 31]. In the present study, patients who had a labial bony defect mesiodistally greater than the implant diameter or vertically more than 4mm from the implant platform level were excluded.

The influence of the implant–socket gap size or the initial facial bone thickness in the case of immediate implant placement is contentious. Chen et al. and Chappuis et al. have reported that sites with thinner post-extraction facial bone underwent significantly more bone resorption than sites with thicker facial bone [32, 33]. The initial bone width facially to the implant is consistent with past reports that facial bone thickness is less than 1.0mm in most cases [28]. Chen also has reported that if the gap width of the extraction socket is 2 mm or less, the interior of the gap will be filled with new bone [34]. In the present study, the horizontal implant-socket gap size was 1.89mm. This result suggests that implants were placed in an ideal position that could keep the proper size of implant-socket gap. Under this criterion, sufficient bone thickness of approximately 2.0mm could be acquired by immediate implant placement with simultaneous GBR facially to the implant during a 1-year follow-up period.

This study supports a past report that found facial bone resorption cannot be wholly prevented even if bone augmentation is combined with immediate placement [3, 11]. However, concerning horizontal thickness, facial bone resorption is compensated for by soft tissue gain after CTG, and the preoperative mucosal contour is maintained. Thus, when the need to preserve the preoperative mucosal contour is not high, as in patients with defects of contralateral teeth, it is considered possible to acquire an esthetically satisfactory outcome without CTG. However, in cases in which a contralateral tooth is present (such as in patients with a single tooth defect), and mucosal contour symmetry between the peri-implant mucosa and the gingival contour of the contralateral tooth is required, CTG should be performed with immediate implant placement to maintain the preoperative mucosal contour.

In this surgical procedure, immediate implant placement and delayed provisionalization were applied to patients, so a mucoperiosteal flap was raised, and sutures were placed to achieve primary wound closure at the time of implant placement. Immediate placement and provisionalization with a flapless technique have been suggested to decrease the amount of soft tissue recession and the risk of wound failure after implant placement [35, 36]. On the other hand, immediate implant placement with delayed restoration is a well-established procedure, especially when the facial bone to implant has dehiscence [30, 37]. This study also included patients with labial bony defects, so contour augmentation should be performed simultaneously with implant placement. Surgical flaps were elevated to allow for GBR procedures to be performed to standardize surgery in this study.

The following limitations of this study should be taken into consideration. First, vertical bone and soft tissue changes were not evaluated in this study. This was because the vertical height of the soft tissue will be positively changed when the implant prosthesis is connected or the emergence profile or facial contour of the provisional restoration is modified. For this reason, it is challenging to evaluate vertical peri-implant tissue changes with the same criteria preoperatively and after connection of the implant prosthesis. Second, the impact of the implant site (central or lateral incisor) or preoperative facial bone condition (presence or size of labial bony defect around the failing tooth) was not explored. If the influence of these factors is to be clarified, future studies with larger samples must be carried out for a more detailed evaluation. Third, only the evaluation 1 year after prosthesis connection has been performed. It has been reported that immediate placement can cause soft tissue recession after treatment and continue for an extended period, up to 5 years after implant placement [5, 38]. A more extended observation period will be necessary to evaluate the grafted peri-implant tissue’s stability and marginal bone levels over time. Forth, in this study, two surgeons decided whether to perform CTG (discrimination of the patients between test and control groups) based on the clinical status of each patient such as mucosal contour compared to the contralateral tooth or gingival biotype. The baseline patient characteristic and initial tissue thickness in the two groups are described in Table 1; there were no statistically significant differences between the groups. However, this study is not based on the randomized selection, and that is one of the limitations of this study. Finally, an esthetic evaluation such as the pink esthetic score was not performed in this study. CTG is beneficial for preserving preoperative mucosal contour, but it is not easy to correlate with esthetic achievement.

In this study, the use of CTG in immediate implant placement was found to be an effective treatment option in the maxillary anterior zone compensating for bone resorption and preserving the preoperative mucosal contour.

In conclusion, facial hard tissue resorption was found in immediate placement sites with GBR only. However, immediate placement sites with GBR and CTG showed evidence of compensation for bone resorption occurring after implant placement with a 1.37 mm soft tissue gain, thus still capable of preserving the preoperative mucosal contour 1 year after connection of the implant prosthesis.

## Data Availability

All the data supporting our findings are in the “Methods” and “Results” sections of this manuscript.
